# Risk Factors and Outcomes in Patients With Acute Exacerbation of Chronic Obstructive Pulmonary Disease

**DOI:** 10.7759/cureus.90919

**Published:** 2025-08-25

**Authors:** Hafiz Muhammad Husnain Shafi, Bilawal Ali, Fahad Dayam, Mudassir Raza, Aymen Bader, Hamna Jabeen Ashraf, Huda Tarique

**Affiliations:** 1 Internal Medicine, Mayo Hospital, Lahore, PAK; 2 Internal Medicine, Allama Iqbal Teaching Hospital, Dera Ghazi Khan, PAK; 3 Internal Medicine, Peshawar Medical College, Peshawar, PAK; 4 Internal Medicine, Sheikh Zayed Hospital, Rahim Yar Khan, PAK; 5 Internal Medicine, Shalamar Hospital, Lahore, PAK

**Keywords:** acute exacerbation, chronic obstructive pulmonary disease, dyspnea, smoke, ventilation

## Abstract

Introduction

Acute exacerbation of chronic obstructive pulmonary disease (AECOPD) is a common cause of hospitalization and is associated with significant morbidity, mortality, and healthcare burden.

Objectives

To determine the risk factors and in-hospital outcomes in patients admitted with acute exacerbation of COPD.

Methodology

This retrospective analysis was conducted at Mayo Hospital, Lahore, Pakistan, from January 2022 to January 2025. A total of 355 patients were enrolled using a non-probability consecutive sampling technique. Demographic details (age, gender, smoking status, residence), clinical history (duration of COPD, previous exacerbations, comorbidities such as diabetes and hypertension), presenting symptoms, and initial investigations, including arterial blood gases, complete blood count, chest X-ray, and C-reactive protein, were recorded.

Results

The mean age of patients was 64.8 ± 9.5 years, with a male predominance of 226(63.7%). A majority, 271(76.3%), were chronic smokers. Common comorbidities included hypertension 148(41.7%), diabetes mellitus 104(29.3%), and ischemic heart disease (IHD) 47(13.2%). Non-invasive ventilation was required in 137(38.6%) of cases, while 48(13.5%) needed invasive mechanical ventilation. ICU admission was required in 67(18.9%) of patients. In-hospital mortality occurred in 34(9.5%) of patients. Significant predictors of poor outcomes included older age (>70 years), IHD, high baseline dyspnea score, and requirement for mechanical ventilation (p < 0.05).

Conclusion

AECOPD leads to high hospitalization and mortality rates, particularly among elderly patients and those with comorbidities like IHD and diabetes. Identifying modifiable risk factors can help improve survival and reduce complications in these patients.

## Introduction

Chronic obstructive pulmonary disease is an advanced and mostly incurable respiratory disease, which is characterized by reduced air circulation. The disease continues to be one of the leading causes of morbidity and mortality in many countries across the world and presents a high burden to the health care system, particularly in low and middle-income economies [1]. According to the World Health Organization, it is estimated that over hundreds of millions of people around the world have chronic obstructive pulmonary disease (COPD), and this is likely to be the third leading cause of death. This is further complex in developing countries like Pakistan by underdiagnosis, less affordability to pulmonary tests, and persistent exposure to risk factors such as tobacco smoke, biomass fuel, environmental pollutants, and weak air quality [2]. Acute exacerbation is regarded as one of the most important complications in the natural history of COPD. Acute exacerbation of chronic obstructive pulmonary disease (AECOPD) is a persistent aggravation of respiratory symptoms that is above the usual daily changes, and frequently requires hospitalization or an intensification of therapy [3]. These incidents correlate with the faster rate of lung functional decline, quality of life diminishment, high hospital admission levels, and high chances of death. Every exacerbation reduces the baseline pulmonary capacity and results in a considerable influence on the long-term disease progression course [4].

Bacterial or viral respiratory infections, contact with environmental irritants, failure to comply with maintenance medications, or illnesses that make existing ones worse, like heart failure or thromboembolism, are some of the causes of exertion. Such triggers are important in terms of prevention and treatment [5]. The problem is that presentations are different, and each episode is multifactorial. Exacerbations are often inadequately managed, leading to recurrent hospitalizations and a progressive deterioration in patients’ overall health status [6]. Some of the most important interrelated clinical factors that characterize an outcome after an exacerbation are severity of air flow obstruction, existence of comorbidity, demographic factors like age and sex, smoking status, nutritional status, history of hospitalizations, and requirements of ventilatory support [7]. Disease severity and prognosis can also be determined with biomarkers; one of them is the elevated C-reactive protein levels and arterial blood gas disturbances. Non-invasive or invasive mechanical ventilation is mainly linked to the long hospital stay, high rates of healthcare expenditure, and likelihood of mortality [8].

Other literature in the recent past has also indicated the role of socioeconomic factors in the management and prognosis of COPD. Lower-income patients will be more inclined to seek delayed care and have a higher presence of barriers when accessing medications and pulmonary rehabilitation [9]. In addition, the pattern of symptom perception and care-seeking behavior has been noted between the genders, and it might impact the clinical outreach [10]. A number of risk stratification clinical tools have been established aimed at screening patient risk, including the BODE index and the DECAF score, which include variables such as body mass index, airflow obstruction, dyspnea, exercise tolerance, eosinophil count, and the presence of comorbidities. Such scoring systems are useful but have not been broadly tested out locally, and their normal application tends to be weak in most state hospitals [11].

Objective

This study was conducted to determine the risk factors and in-hospital outcomes among patients admitted with AECOPD.

## Materials and methods

Methodology

This retrospective analysis was conducted at Mayo Hospital, Lahore, Pakistan, from January 2022 to January 2025. A total of 355 patients were enrolled using a non-probability consecutive sampling technique.

Inclusion and exclusion criteria

Patients aged 40 years and above who were clinically diagnosed with COPD according to the GOLD criteria and presented with an acute exacerbation, defined as a worsening of respiratory symptoms requiring a change in regular medication or hospitalization, were included in the study. Patients were excluded if they had other primary pulmonary conditions such as asthma, tuberculosis, or lung cancer; were hemodynamically unstable due to other acute conditions such as acute myocardial infarction or cerebrovascular accident (CVA); or had incomplete medical records.

Data collection

Demographic details (age, gender, smoking status, residence), clinical history (duration of COPD, previous exacerbations, comorbidities such as diabetes and hypertension), presenting symptoms, and initial investigations, including arterial blood gases, complete blood count, chest X-ray, and C-reactive protein, were recorded. The need for non-invasive or invasive ventilation, length of hospital stays, ICU admission, and in-hospital mortality were also documented.

Statistical analysis

Data were entered and analyzed using SPSS version 26. Quantitative variables such as age and hospital stay were presented as mean ± standard deviation (SD) and compared using the independent t-test. Categorical variables, including gender, smoking history, comorbidities, and ventilation requirements, were expressed as frequencies and percentages, and associations were assessed using the chi-square test. Predictors of in-hospital mortality were evaluated through binary logistic regression analysis, with results presented as odds ratios (OR) with 95% confidence intervals (CI). A p-value < 0.05 was considered statistically significant.

## Results

Out of 355 patients, the mean age was 64.8 ± 9.5 years. There were 226 males (63.7%) and 129 females (36.3%). A majority were current or ex-smokers (271, 76.3%), while 84 (23.7%) were non-smokers. Hypertension was present in 148 patients (41.7%), type 2 diabetes in 104 (29.3%), and ischemic heart disease (IHD) in 47 (13.2%). The most common symptoms were worsening dyspnea in 326 patients (91.8%), increased sputum in 279 (78.6%), and elevated temperature in 222 (62.5%) (Table 1).

**Table 1:** Demographic and Clinical Characteristics of Study Participants (n = 355) Data are presented as Mean ± SD for continuous variables and N (%) for categorical variables. No hypothesis testing was applied in this descriptive table.

Characteristic	Value
Mean Age (years)	64.8 ± 9.5
Gender	
- Male	226 (63.7%)
- Female	129 (36.3%)
Smoking Status	
- Current/Ex-smoker	271 (76.3%)
- Non-smoker	84 (23.7%)
Comorbidities	
- Hypertension	148 (41.7%)
- Type 2 diabetes mellitus	104 (29.3%)
- Ischemic heart disease	47 (13.2%)
Presenting Symptoms	
- Worsening dyspnea	326 (91.8%)
- Increased sputum	279 (78.6%)
- Elevated temperature	222 (62.5%)

Non-invasive ventilation was used in 137 patients (38.6%) and invasive mechanical ventilation in 48 (13.5%). ICU admission occurred in 67 cases (18.9%), and the mean hospital stay was 6.4 ± 2.9 days. A total of 291 patients (82%) recovered without complications, while 30 (8.5%) had prolonged hospitalization exceeding 10 days. In-hospital mortality was documented in 34 patients (9.5%), reflecting the severity of illness in this cohort (Table 2).

**Table 2:** Hospital Management, Interventions, and Outcomes (n = 355) Data presented as Mean ± SD or N (%). p-values calculated using the chi-square (χ²) test. Significance is defined as p < 0.05.

Intervention/Outcome	Value (N, %)	Test Statistic	p-value
Non-Invasive Ventilation (NIV)	137 (38.6%)	χ² = 14.82	< 0.001
Invasive mechanical ventilation	48 (13.5%)	χ² = 24.61	< 0.001
ICU admission	67 (18.9%)	χ² = 6.29	0.012
Mean hospital stay (days)	6.4 ± 2.9	—	—
Recovered without complications	291 (82.0%)	—	—
Prolonged hospitalization (>10 days)	30 (8.5%)	χ² = 4.79	0.028
In-hospital mortality	34 (9.5%)	χ² = 18.07	< 0.001

Of the 355 patients, 321 (90.5%) survived, while 34 (9.5%) died during hospitalization. Non-survivors were significantly older compared to survivors (70.5 ± 8.6 vs. 64.1 ± 9.3 years, p = 0.002). IHD was also more prevalent among non-survivors (26.5% vs. 11.8%, p = 0.04). Invasive mechanical ventilation was required far more frequently in non-survivors (41.2% vs. 10.6%, p < 0.001), and ICU admission was also higher in this group (41.2% vs. 16.5%, p = 0.001). Although hypertension, type 2 diabetes mellitus, and smoking were more common in non-survivors, these differences did not reach statistical significance. The mean hospital stay was slightly longer among non-survivors (7.1 ± 3.2 vs. 6.3 ± 2.8 days), though this difference was not significant (p = 0.12) (Table 3).

**Table 3:** Comparison of Survivors vs. Non-Survivors in AECOPD (n = 355) Data are presented as Mean ± SD for continuous variables and N (%) for categorical variables. An independent-samples t-test was applied for continuous variables, and a chi-square (χ²) test for categorical variables. A p-value < 0.05 was considered statistically significant. AECOPD : Acute exacerbation of chronic obstructive pulmonary disease

Characteristic	Survivors (n=321)	Non-survivors (n=34)	Test Statistic	p-value
Mean Age (years)	64.1 ± 9.3	70.5 ± 8.6	t = -3.16	0.002
Male gender	202 (62.9%)	24 (70.6%)	χ² = 0.78	0.38
Smoking (current/ex)	241 (75.1%)	30 (88.2%)	χ² = 2.87	0.09
Hypertension	130 (40.5%)	18 (52.9%)	χ² = 1.91	0.17
Type 2 diabetes mellitus	91 (28.3%)	13 (38.2%)	χ² = 1.50	0.22
Ischemic heart disease	38 (11.8%)	9 (26.5%)	χ² = 4.11	0.04
Non-invasive ventilation	126 (39.3%)	11 (32.4%)	χ² = 0.59	0.44
Invasive ventilation	34 (10.6%)	14 (41.2%)	χ² = 22.64	<0.001
ICU admission	53 (16.5%)	14 (41.2%)	χ² = 10.24	0.001
Mean hospital stay (days)	6.3 ± 2.8	7.1 ± 3.2	t = -1.55	0.12

Invasive mechanical ventilation significantly predicted mortality, with an OR of 4.63 (95% CI: 2.10-10.21; p < 0.001). IHD was also a significant predictor, with an OR of 2.14 (95% CI: 1.03-4.43; p = 0.04) (Table 4).

**Table 4:** Significant Predictors of In-Hospital Mortality in Acute exacerbation of chronic obstructive pulmonary disease (AECOPD) Patients Odds Ratios with 95% Confidence Intervals are reported. p-values computed using the chi-square test. Significance threshold set at p < 0.05.

Predictor	Odds Ratio (OR)	95% CI	Test Statistic (χ²)	p-value
Invasive ventilation	4.63	2.10 – 10.21	χ² = 22.64	< 0.001
Ischemic heart disease	2.14	1.03 – 4.43	χ² = 4.11	0.04

**Figure 1:**
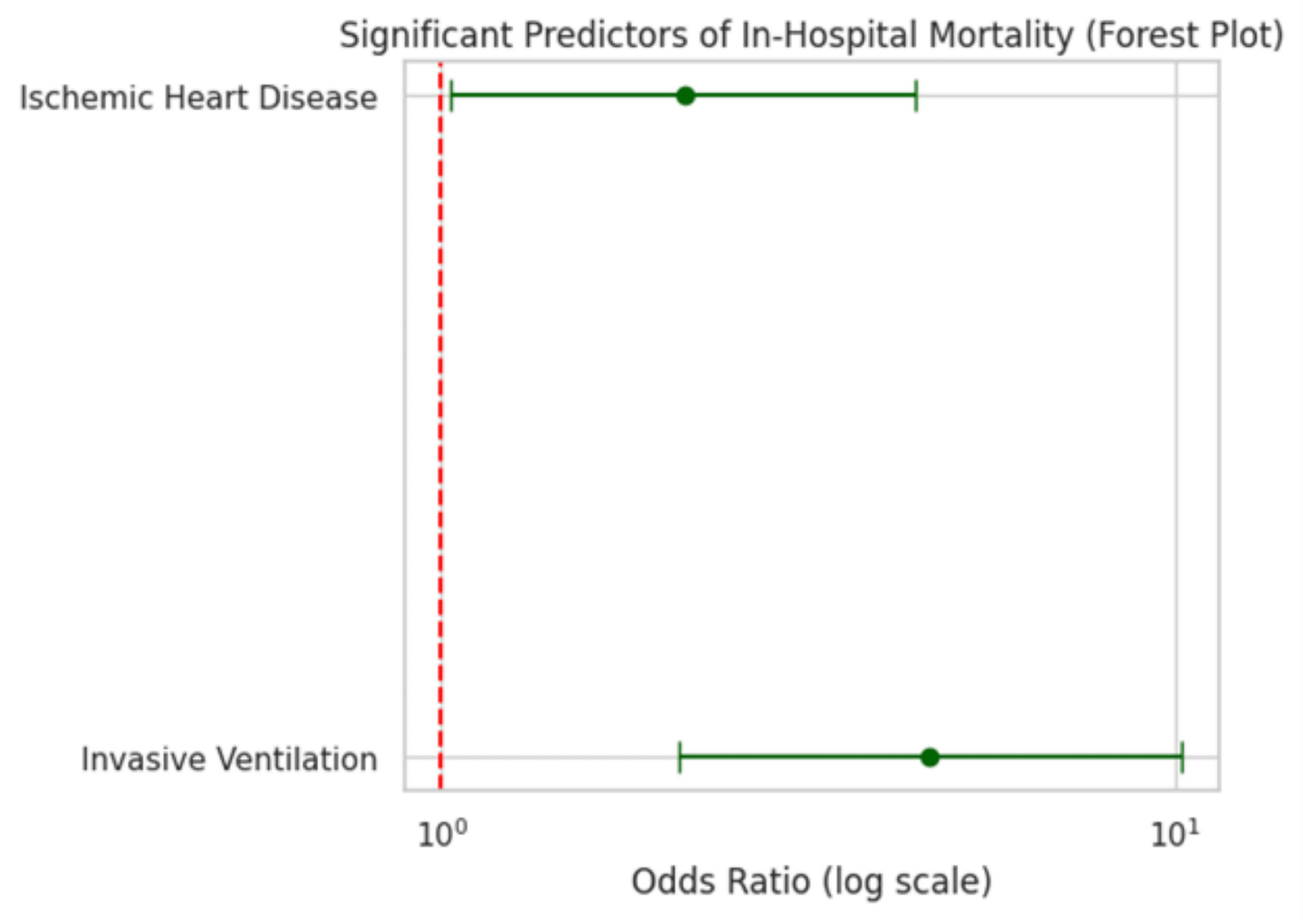
Forest Plot Showing Significant Predictors of In-Hospital Mortality in COPD Patients with Acute Exacerbation Predictors: Invasive Ventilation, Ischemic Heart Disease ORs (95% CI): Invasive Ventilation: OR = 4.63 (CI: 2.10–10.21), χ² = 22.64, p < 0.001 IHD: OR = 2.14 (CI: 1.03–4.43), χ² = 4.11, p = 0.04 COPD: chronic obstructive pulmonary disease; IHD: ischemic heart disease The forest plot shows odds ratios and 95% confidence intervals for significant predictors of in-hospital mortality. p-values calculated via chi-square test. Significance set at p < 0.05.

The forest plot shows that invasive ventilation (OR ≈ 4.63, CI: 2.10-10.21) and IHD (OR ≈ 2.14, CI: 1.03-4.43) are significant predictors of in-hospital mortality. Both confidence intervals lie entirely to the right of OR = 1, indicating increased risk (Figure 1).

## Discussion

This paper sought to discuss clinical profile, treatment, and outcomes of patients who were admitted to the hospital because of AECOPD, and to highlight some of the main risk factors related to poor outcomes, especially mortality in the hospital. We present a virtual panorama of the AECOPD burden in tertiary care and reveal a number of patterns in accordance with the previous evidence base on a global and regional scale. The average age of the participants comprising our study group was 64.8 years, which fits previously defined information that AECOPD is a phenomenon specific to the elderly population, given that chronic exposure to cigarette smoking and aging-induced functional deterioration in lungs go hand in hand [12]. There was male predominance, 226 (63.7%), as is known in developing countries that have the higher prevalence of smoking and work-related exposures. Nonetheless, the emerging trend in increased prevalence of smoking among females is a factor that is likely to alter such a pattern in the future [13].

A significant majority of patients, 271 (76.3%), were current or former smokers, making the central role of tobacco exposure in the pathogenesis of COPD again. Duplicate results were identified by the analysis published in both the high-income and low- to middle-income countries, with research indicating an extreme necessity of tobacco-quitting interventions to prevent both the progression of the disease and its exacerbation [14]. Comorbidities were common whereby 148 (41.7%) were hypertensive, 104 (29.3%) diabetic, and 47 (13.2%) had IHD. This can be matched to the logistics of prior research, which revealed that multiple chronic conditions are common among patients with COPD because of the common risk factors, which include age, smoking, and patients with systemic inflammation [15]. Of importance, IHD is another finding of our research that served as a crucial predictor of mortality (OR: 2.14, p = 0.04). This fact explains why efforts should be made to control cardiovascular risk in COPD patients aggressively. Worsening dyspnea 326(91.8%) and increased sputum production 279 (78.6%) prevailed and constitute touched upon cardinal features of exacerbation according to GOLD guidelines [16]. Nearly half of them also reported febrile illness 222 (62.5%), which designates probable infectious factors or causes, which are also well-reported triggers of AECOPD. From a management perspective, 137 (38.6%) of patients were treated using non-invasive ventilation, with 48 (13.5%) developing invasive mechanical ventilation [17]. These statistics reflect earlier reviews that indicated that early intervention of NIV using the device could minimize the necessity of intubation and improve results. Patients who sought invasive ventilation, though, had a much greater chance of mortality (OR: 4.63, p < 0.001), which indicates the drastic character of respiratory failure in this case and its unfavorable outcomes [18]. The total in-hospital mortality rate accounted for 34 (9.5%), and it is not beyond the range featured by other similar studies, with rates typically varying from 5% to 15% [19]. The majority, 291 (82%) of the patients recovered without complications, and 30(8.5%) of the patients had a longer hospital stay period (>10 days) [20,21]. These results suggest that with early and appropriate inpatient treatment, many AECOPD cases have a favorable prognosis, although a significant minority may still experience complications or mortality.

Despite several strengths, including a relatively large sample size and comprehensive clinical data, this study has notable limitations. First, as a single-center retrospective analysis, the findings may not be generalizable to other populations or healthcare settings. Second, the absence of post-discharge follow-up precludes assessment of long-term outcomes, such as readmissions or mortality beyond hospitalization. Third, important potential confounders, including medication adherence, socioeconomic status, and pulmonary function test results, were not captured, which may influence some associations. Fourth, the use of non-probability consecutive sampling could introduce selection bias, as the included patients may not fully represent the broader COPD population. Fifth, although inflammatory markers such as C-reactive protein (CRP) were recorded, their prognostic value was not statistically analyzed, and other biomarkers (e.g., procalcitonin, eosinophil counts) were inconsistently available. Finally, model performance metrics such as AUC/ROC were not calculated, and effect size reporting beyond ORs was limited, which restricts the assessment of the predictive strength of identified risk factors.

## Conclusions

It is concluded that acute exacerbation of COPD remains a significant cause of hospitalization and morbidity, particularly among elderly patients, males, and long-term smokers. Comorbidities such as IHD and the need for invasive mechanical ventilation were strongly associated with poor outcomes, including increased in-hospital mortality. Despite appropriate inpatient management, nearly one in ten patients succumbed to complications during hospitalization. Timely recognition of risk factors, especially cardiovascular comorbidities and respiratory failure, is essential to improve clinical outcomes. Preventive strategies, including optimization of comorbid conditions, timely escalation of respiratory support, and structured ICU admission protocols, may help mitigate poor outcomes. Further multicenter prospective research is recommended to validate these predictors and to explore additional risk factors, such as diabetes mellitus, that were not specifically analyzed in the present study but are known from prior literature to influence prognosis.
